# A Figure One Web Tool for Visualization of Experimental Designs

**DOI:** 10.5334/jors.243

**Published:** 2020-03-30

**Authors:** Foo Cheung, The CHI Consortium

**Affiliations:** 1 NIH, US; 2 NIH (Yuri Kotliarov, Julián Candia, Katherine Stagliano, Angélique Biancotto and John S. Tsang), US

**Keywords:** Experimental Design, summary figures, information visualisation tool, Shiny, experimental studies

## Abstract

This manuscript introduces a user-friendly, point and click open source and platform-independent software tool that aids the graphical representation of experimental studies. A graphical summary can give a high-level view of a study and represent in one illustration the important features of the data. Examples include sample collections, the time of each data collection, perturbations, and analysis performed. Graphical summaries can be useful in clarifying and documenting the complex relationships within an experiment by breaking down the component parts and expressing them visually. Commonly used cases for this tool include generating summary figures for presentation and publications. This tool was used either alone or in conjunction with other tools to generate schematic diagrams for talks and publications on several different on-going research projects.

## (1) Overview

### Introduction

Experimental designs can be both complex and time-consuming. However, they provide useful information by improving transparency and documenting key experimental steps such as the collecting and the analysis of large datasets using different tests over time where subjects or animals are exposed to certain treatments [1, 2, 3, 4].

Currently, there is a variety of drawing programs that can be used to draw diagrams, including Gimp (https://www.gimp.org/), Inkscape (https://inkscape.org/en/), PowerPoint (https://products.office.com/en-us/powerpoint) and TikZ (https://sourceforge.net/projects/pgf/).

Experimental studies involving multiple tests, perturbation (disturbance on the biological system that causes it to change e.g.: Vaccination) and timepoints (points in time) can be complex and error prone to follow without the aid of an experimental design. We have seen a need for a web tool that can quickly generate schematic diagrams in a point and click manner. Here we present such a tool that avoids time-consuming tasks like drawing circles or arrows using primitive tools. This tool has already been successfully used to generate numerous experimental designs from several on-going research projects.

Although there is a minimal learning curve and an investment in development time, this tool was created with the aim of being easy to use, highly portable and containing useful features. There is not a method to hand-draw shapes, this tool is focused on specifying the exact locations of text (e.g.: labels, Timepoints, titles etc), lines, arrows and shapes by selecting coordinates. There is no programming functionality but the user is able to run loops that creates a series of lines, arrows, shapes with a varying parameter by selecting x-coordinates (time points) and y-coordinates.

The first step towards creating an experimental design requires the user to decide on whether to start from scratch or from one of the available templates.

### Creating a Diagram

There are several options available in order to create a schematic of an experimental design. The first method involves using a pre-existing template, and second method involves using a blank canvas.

### Using a Template

**Figure 1:**
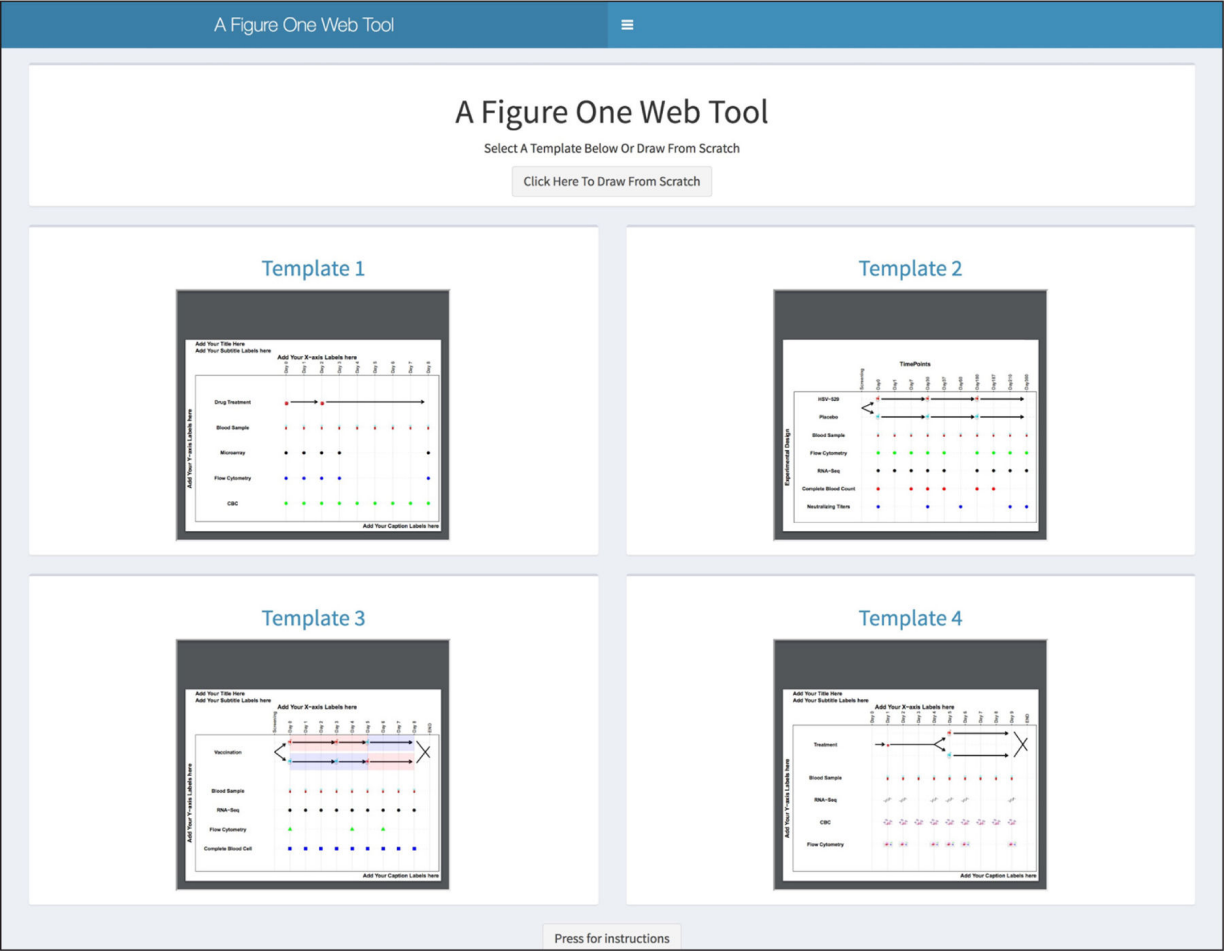
Creating a diagram based upon a pre-existing template.

Our tool allows a user to create a diagram based on a pre-existing template (Figure 1), then edit and add your own content which can save time and effort. The user can start a new diagram by editing an existing template, which consists of a URL that specifies the styles, settings, and layouts. The template can also serve as a useful reminder on how to create certain designs. Custom templates can be created by copying the URLs which then can be saved, reused and shared with collaborators. A selection of templates is available on the landing page. Click on the template link above each template that you would like to open and then navigate to the template window on your computer. Once the web page loads, the user would click the ‘Draw Figure!’ button to generate the visualization and then start editing. It is unlikely that you will find a template that precisely matches your needs, but you can select the closest fit and then modify it.

### Creating a Blank Canvas

**Figure 2:**
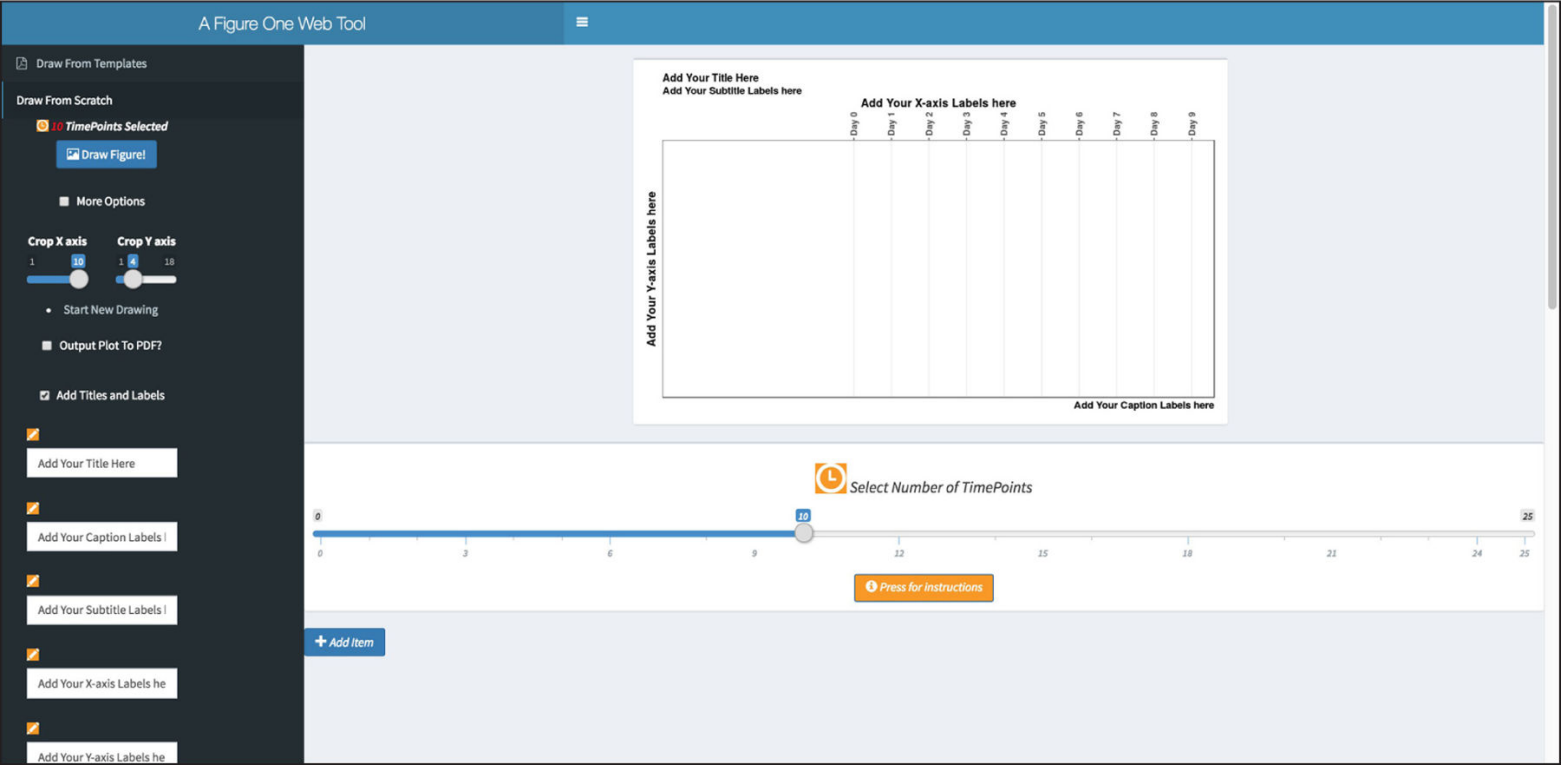
Creating a diagram from scratch.

The user starts by setting up the initial scope of the framework by selecting the total number of time points. Once the time points are selected the user can browse a library of shapes. The shapes and coordinates now represent the relationships between key steps such as the blood draws, time points, vaccinations, medication, transcriptomics, proteomics, etc. The first step requires the user to select and click on the ‘Draw from Scratch’ button or link to start a new visualization. Click on the button ‘Draw Figure!’ to create a blank canvas with a placeholder for the title, sub-title, captions, timepoints, x and y labels as shown in Figure 2, default values for Timepoints are initially set to ten Timepoints.

### Creating Content

Users can select the number of timepoints or columns with the central slider. If a user needs to make adjustments, they can add or delete timepoints by moving and adjusting the slider.

Titles and labels can be added to the canvas by completing the automatically generated text boxes, found within the navigation side bar or remove the default text to leave it blank.

Click on ‘Add Item’ to add content. The user can place multiple shapes, icons and images by specifying precise coordinates which will reflect the locations across columns and rows on the canvas. Content can include different colours and sizes of arrows, circles, boxes, images, icons and rectangles. Rows and column coordinates will decide the location of content on a canvas and pressing the button ‘Draw Figure!’ will update the diagram.

### Proof of Concept

**Figure 3:**
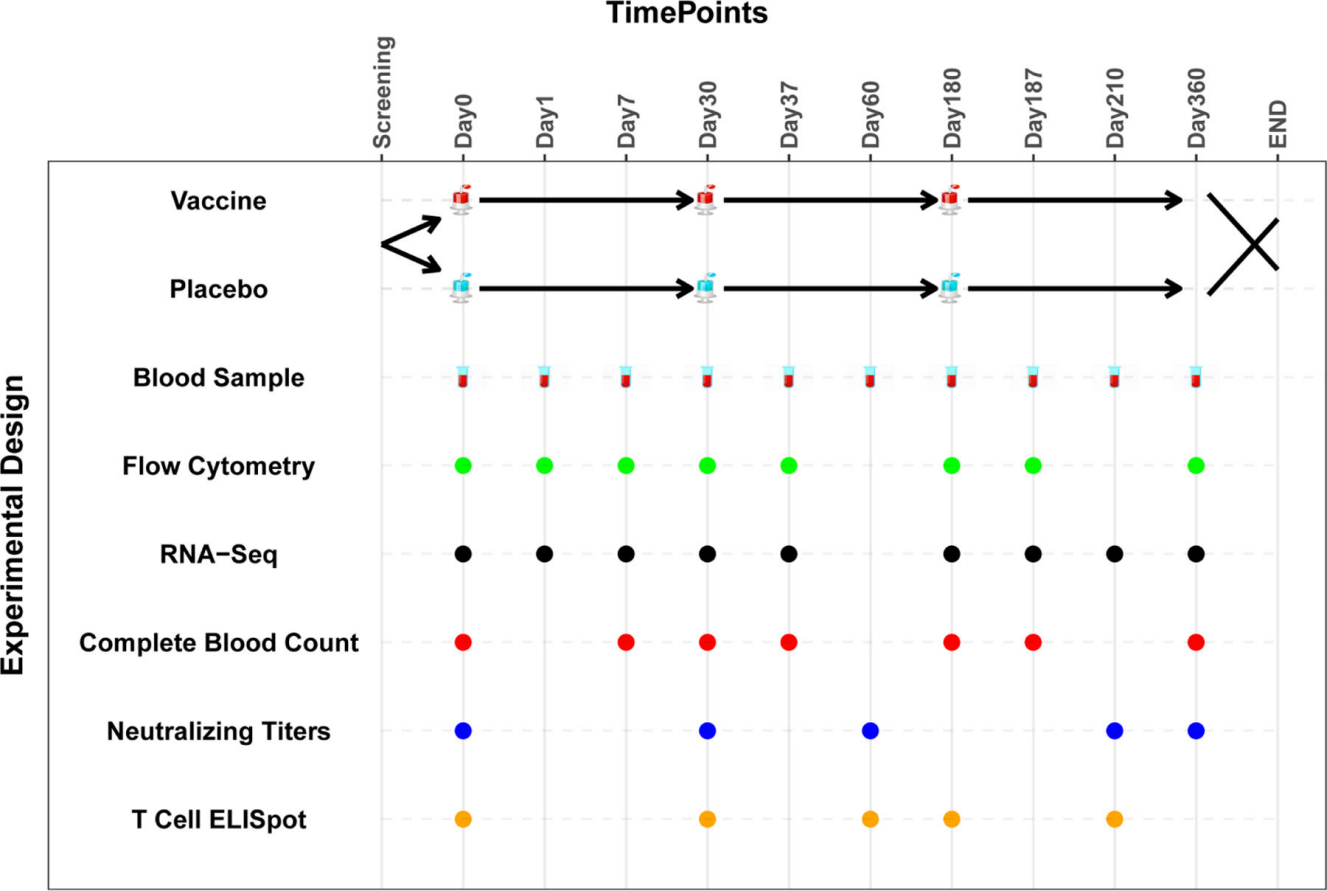
An example of a diagram output.

As proof of concept we used the web tool to create a schematic diagram based on an actual experimental design for a clinical trial as shown in Figure 3.

### Output As PDF

Click on the “Output Plot To PDF” and follow the download link to a high resolution of your illustration.

### Bookmark and Helpful Hints

The best way to avoid losing work is to save the URL early and save often. The bookmark feature allows the user to recreate the diagram from the URL and enables sharing with collaborators. We also implemented a dynamic interactive help system by clicking on the “Press for instruction button” which will then walk through and explain each feature.

### Implementation and architecture

This web application is written using the shiny framework [5], a package from RStudio that can be used to build interactive web pages with R [6]. The code is split into two parts, (1) the user interface and (2) the server-side containing the logic. Numerous R packages were used including shinydashboard [7], ggplot2 [8]. See code for complete list.

### Quality control

The software has been through several rounds of functional and usability testing. Each feature is tested by typing the input and examining the output making sure it operates according to the requirement specification. Usability testing was carried out by user feedback and manually checking completed diagrams. We have successfully created numerous diagrams with this tool to date. Support through this web tool is by feedback, bug reports and feature wishes from numerous users. The application runs in most modern web browsers, including Google Chrome, Safari, Firefox, and IE10+ and several operating systems, including Windows, Mac, Linux and Chrome.

## (2) Availability

### Operating system

A platform-independent software package, compatible with modern web browsers (IE10+, Google Chrome, Firefox, Safari, etc.).

### Programming language

R

### Additional system requirements

None.

### Dependencies

Imports a number of R packages (see code for most up-to-date list).

### Installation

We expect that most users will use the web tool directly from the website https://github.com/foocheung/figureone/blob/master/README_deploy.txt but users can also install this web tool from source code https://github.com/foocheung/figureone if required.

### List of contributors

NA

### Software location

#### Code repository

***Name:*** GitHub

***Identifier:*** https://github.com/foocheung/figureone

***License:*** Apache

***Date published:*** 08/02/2018

### Language

R

## (3) Reuse potential

As a standalone program, this web tool provides researchers with a way to draw experimental designs or summaries which in an intuitive, ‘clickable’ manner. This program has a high reuse potential “as is” and has already been a useful tool for several clinical projects from a wide spectrum of biology regardless of coding experience.
